# Heterogeneity assessment of functional T cell avidity

**DOI:** 10.1038/srep44320

**Published:** 2017-03-13

**Authors:** Kalliopi Ioannidou, Petra Baumgaertner, Philippe O. Gannon, Michel F. Speiser, Mathilde Allard, Michael Hebeisen, Nathalie Rufer, Daniel E. Speiser

**Affiliations:** 1 Ludwig Cancer Research Center, University of Lausanne, Lausanne, Switzerland; 2 Department of Oncology, Lausanne University Hospital Center (CHUV), University of Lausanne, Lausanne, Switzerland; 3 IBM Research - Zurich, Rueschlikon, Switzerland

## Abstract

The potency of cellular immune responses strongly depends on T cell avidity to antigen. Yet, functional avidity measurements are rarely performed in patients, mainly due to the technical challenges of characterizing heterogeneous T cells. The mean functional T cell avidity can be determined by the IFN-γ Elispot assay, with titrated amounts of peptide. Using this assay, we developed a method revealing the heterogeneity of functional avidity, represented by the steepness/hillslope of the peptide titration curve, documented by proof of principle experiments and mathematical modeling. Our data show that not only natural polyclonal CD8 T cell populations from cancer patients, but also monoclonal T cells differ strongly in their heterogeneity of functional avidity. Interestingly, clones and polyclonal cells displayed comparable ranges of heterogeneity. We conclude that besides the mean functional avidity, it is feasible and useful to determine its heterogeneity (hillslope) for characterizing T cell responses in basic research and patient investigation.

The field of oncoimmunology is rapidly evolving, with several key milestones reached lately, resulting in increasing clinical efficacy and enhanced understanding of the role of T cells in anti-tumor immunity. Cancer cells can be recognized by the immune system, and in some cases, the immune system can control or even eliminate tumors^1^,^2^. Both innate and adaptive immunity contribute to the recognition and rejection of malignant cells^3^,^4^. The development of immunotherapy represents one of the most significant advances in the history of cancer therapy. Adoptive T cell transfer^5^,^6^,^7^,^8^ and checkpoint blocking antibodies, specifically antibodies directed against cytotoxic T-lymphocyte antigen 4 (CTLA-4) and programmed death 1 receptor (PD-1) or PD-ligand 1 (PD-L1)^9^ have achieved impressive clinical results and demonstrated significant progress in the treatment of an expanding list of malignancies^10^,^11^. Nonetheless, further hurdles have to be overcome in order to improve the efficacy of immunotherapies for cancer patients. Novel combinatorial immunotherapeutic strategies may enhance the killing capacity of anti-tumor T cells, improve therapeutic modifications of the tumor microenvironment, prevent immune inhibitory mechanisms utilized by tumor cells, and minimize autoimmune and/or toxic side effects.

During an immune response, T cells respond with great sensitivity and selectivity to antigens^12^. More than 20 years ago we pioneered the field by demonstrating that low T cell avidity is sufficient for T cell proliferation and T cell mediated killing *in vitro*, but not for antiviral activity *in vivo*^13^. This principle was confirmed and extended by others^14^ establishing that CD8 T cells with high functional avidity show better protection *in vivo* than their low avidity counterparts^15^,^16^. Importantly, the functional avidity of T cells is guided by the binding strength of the T cell receptor (TCR) to cognate antigen, i.e. the peptide/major histocompatibility class I (pMHC) complex. Indeed, the TCR-pMHC affinity/avidity plays multiple crucial roles in positive/negative selection, induction of anergy/tolerance, triggering of autoimmune disease and control of infections and cancers^17^,^18^,^19^. In cancer, CD8 T cells often recognize tumor-associated antigens of self-origin. Due to thymic negative selection, tumor-specific T cells express TCRs of usually lower affinity/avidity compared with TCRs specific for microbial (nonself) epitopes^20^,^21^. Therefore, to evaluate the potency of anti-tumor T cells, it may be particularly important to determine their functional avidity.

The strength of the TCR/pMHC interaction on the cell surface is primarily determined by the TCR affinity, which defines the physical strength of the monomeric interaction between a single TCR with its cognate pMHC complex, as assessed by surface plasmon resonance technique^22^,^23^,^24^. In addition, several factors that are independent of TCR affinity regulate functional avidity of T cells, such as TCR clustering, involvement of the co-receptor CD8, adhesion molecules, and co-activating and co-inhibitory receptors/ligands^25^ or recognition efficiency^3^,^26^,^27^. The mean functional avidity is assessed by cellular assays such as the ^51^Chromium release cytotoxicity assay^28^,^29^ or the IFN-γ Elispot assay^30^, using titrated amounts of peptide.

Despite its importance, the role of avidity of tumor-antigen specific CD8 T cells in cancer patients remains largely unknown. Current immunomonitoring techniques are insufficient with regard to the assessment of T cell avidity and TCR affinity. Flow cytometry using tetramers reveals frequencies and functionality of T cells, but most often without data on T cell avidity. Recently, elegant studies using a Streptamer-based assay^31^ or the NTA-multimer technology^21^,^32^, have allowed the direct and precise quantification of TCR: pMHC dissociation rates (*k*_off_) on living CD8 T cells, demonstrating that the k_*off*_ rate correlated with the functional and protective capacity of antigen specific CD8 T cells. In addition, two-dimensional (2D) measurements of TCR-pMHC interactions provide novel opportunities to characterize T cell affinity and antigen specificity^33^. Combining functional with structural avidity assessments enables detailed characterization of specific T cell populations^34^. However, the available techniques are primarily designed for monoclonal T cells, whereas the characterization of polyclonal T cell responses remains challenging. In the present study we attempted to evaluate the heterogeneity of T cell avidities, representing an additional parameter for the characterization of T cell responses. We demonstrate a new method to determine the heterogeneity of functional avidity, and characterize the avidity range of T cell clones and related natural polyclonal populations.

## Methods

### Patients

29 HLA-A * 0201–positive patients with stage III/IV metastatic melanoma received series of monthly subcutaneous vaccinations with 0.1 mg Melan-A/MART-1_26–35_ peptide and 0.5 mg CpG 7909/PF-3512676 (Pfizer and Coley Pharmaceutical Group), emulsified in IFA (Montanide ISA-51; Seppic)^35^. The trial (LUD 00-018, ClinicalTrials.gov number NCT00112229, registered May 31, 2005) was conducted according to the relevant regulatory standards, upon approval by Swissmedic (the regulatory agency of Switzerland) and the ethical committee of the University of Lausanne, which also approved the experimental protocols and the use of PBMC from healthy volunteers. All methods were carried out in accordance with relevant guidelines and regulations. Patients were enrolled upon written informed consent.

### Generation of CD8 T cell clones

Blood withdrawal and handling, as well as further details are described in Supplementary Methods. Briefly, CD8+ cells were purified using MS columns, loaded with a maximal number of 10 × 10^6^ PBMCs labeled cells, using the MiniMACS^TM^ separators attached to the MACS MultiStand Magnet (Miltenyi). For generating T cell clones, antigen specific T cells were directly sorted with fluorescent MHC/peptide multimers and CD8 mAb, using a FACS Aria cell sorter (BD Biosciences). Sorted T cells were cloned by plating in standardized culture conditions at the concentration of respectively, 0.5 and 1 cell per well, in Terasaki plates, and stimulated with 1 × 10^6^/ml irradiated allogeneic PBMCs (feeder cells), PHA (1 μg/ml) and IL-2 (150 U/ml).

### Determination of hillslope by a Hill function with variable slope

IFN-γ Elispot assays were performed as described in Supplementary Methods. Decreasing numbers of spots were obtained with titrated (diluted) amounts of peptides. The data were used to draw sigmoidal curves. As widely used, a Hill function with fixed slope (three-parameter Hill equation for sigmoidal standard fit; the log (agonist) vs. response model), represented by Eq. (1),


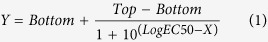


provides a curve with a symmetrical sigmoidal shape. Using this, the parameters Top, Bottom and LogEC_50_ are estimated. A slightly more flexible model, represented by Eq. (2), includes an additional hillslope parameter, which controls the slope of the curve;


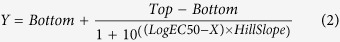


This model is also called a four parameter Hill equation. We used the Eq. (2) “log (agonist) vs. response-variable slope (four parameters)” in Prism, to which we refer hereafter as “Hill function with variable slope”, for addressing functional avidity in clones and in polyclonal populations. In this model, the hillslope describes the steepness of the curve, whereas a standard sigmoidal dose-response curve, Eq. (1), has an implicit hillslope of 1.0. The goodness of the fit is described by the R square value. The data from different clones with different avidities fitted better when the hillslope was unconstrained, according to the adjusted RMSE called “Sy.x” which accounts for the difference in number of parameters. Furthermore, with this function the plateaus (minimal and maximal numbers of spots) are variables. Therefore, there is no need for normalization. Without this, the spot values would need to be normalized according to minimal and maximal numbers of spots, to exclude that elevated background values or low maximal spot numbers impacting improperly on the hillslope value.

### Biphasic equation and supportive mathematical model

In order to more accurately fit the data obtained with mixtures of two clones, a special dose-response was used, the Biphasic dose response model, represented by Eq. (3);


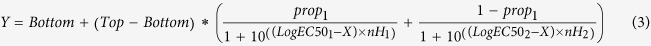


where *prop*_1_ stands for the proportion (between 0 and 1) of clone 1 in the mix. We considered constraining *nH*_1_ (Hillslope of clone 1) and *nH*_2_ (Hillslope of clone 2) to constant values of 1.0. *LogEC*50_1_ and *LogEC*50_2_ correspond to the concentrations that give half-maximal activity of the respective clone. In Prism, this model is available under the name “Biphasic dose-response”. Equation (3) gives rise to a double-S curve, which was empirically observed when the mixing clones had a big EC_50_ difference. Constraining both Hillslopes (*nH*_1_ and *nH*_2_) to 1 allowed direct comparisons of goodness of curve fitting using the R square values that is well suited when the functions have the same numbers of parameters. This function has four parameters (2× EC_50_, Top, Bottom), similar to the Hill function with variable slope (EC50, hillslope, Top, Bottom).

## Results

### Assessment of functional avidity of T cell clones

**Figure 1:**
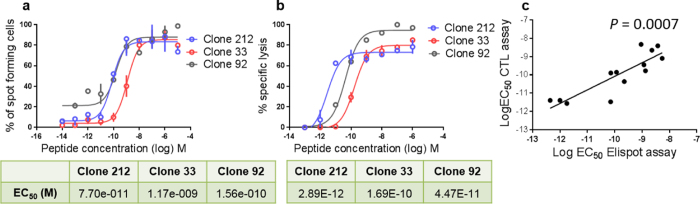
Assessment of the mean functional avidity of T cell clones: comparison of IFN-γ Elispot and cytotoxicity assays. IFN-γ producing T cell clones stimulated with T2 cells and titrated peptide concentrations. (**a**) Elispot data from 3 different clones. 300 Melan-A specific T cell clones were plated together with 20,000 T2 cells per well as described in Supplementary Methods. (**b**) Peptide titration data obtained with the cytotoxicity assay, using the same 3 representative clones. 10,000 monoclonal T cells and 1,000 T2 target cells were plated per well (**c**). Comparison of mean functional avidity (Log EC_50_) of 13 T cell clones, determined with both the Elispot assay (in triplicates) and the cytotoxicity assay. The vertical lines show the standard deviations (SDs) of the triplicates, absence of vertical lines is due to very small SDs.

As many other laboratories, we frequently use the ^51^Chromium release cytotoxicity assay to determine the mean “functional avidity” of cytotoxic CD8 T cells, i.e. the peptide concentration (EC_50_) at which the T cells show 50% of maximal activity. Here, we tested whether the same goal can be achieved by the IFN-γ Elispot assay (as described in Supplementary Methods). In analogy to the cytotoxicity assay, we analyzed IFN-γ production in the Elispot assay with titrated amounts of antigenic peptide to determine the EC_50_ values. Figure 1a shows 3 representative clones with different functional avidities as assessed by the IFN-γ Elispot assay, and Fig. 1b shows the data obtained with the cytotoxicity assay. The results demonstrate a strong correlation between the log EC_50_ values obtained with the two assays (Fig. 1c), indicating that both methods are equally suited for the assessment of the mean functional avidity. However, the Elispot assay requires lower cell numbers than the cytotoxicity assay, making it more attractive for titration experiments and wide spread analysis of precious clinical samples. Therefore, we used this method for the subsequent analyses.

### Different shapes of the titration curves

The standard mathematical assessment classically used to determine the mean functional avidity generates a sigmoid curve (Fig. 1a), drawn by a Hill function with fixed slope. We reasoned that in reality the curves may have different shapes, and were particularly interested in the slope of the curve. Specifically, we hypothesized that the slope of the curve may correspond to the degree of heterogeneity of functional avidity. Theoretically, T cells with completely homogeneous functional avidity should give a vertical curve, whereby all clones spot at exactly the same peptide concentration. In turn, the slope should decrease, i.e. become shallower, with increasing heterogeneity of the functional avidity (illustrated in the Supplementary Fig. 1a and b, respectively).

**Figure 2:**
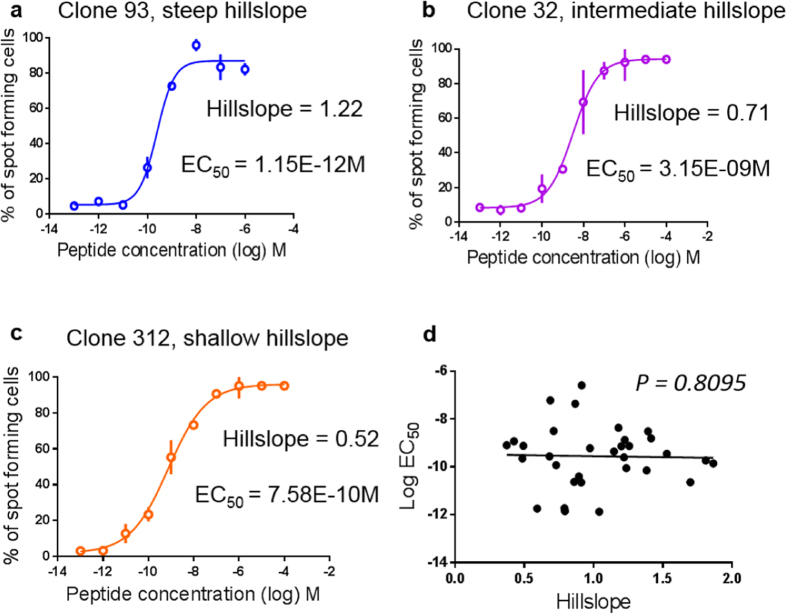
Elispot titration curves from 3 individual T cell clones with different hillslopes, and lack of correlation with EC_50_. (**a**) A T cell clone with a steep hillslope, (**b**) an intermediate hillslope, (**c**) or a shallow hillslope, as determined using the Hill function with variable slope (four parameters), where the dose response curves are defined by these four parameters: EC_50_, hillslope, top plateau and bottom plateau. Data are representative for at least 3 repeat experiments, confirming that not only the EC_50_ values but also the hillslope values were reproducible (not shown). (**d**) EC_50_ and hillslope do not correlate.

Using the Hill function with variable slope, we observed that the slopes of the Elispot titration curves were not the same for the different T cell clones analyzed. Some clones displayed steep curves, while others shallow curves, or curves with intermediate steepness, as illustrated in Fig. 2a–c from 3 representative clones having different hillslopes and different IFN-γ Elispot EC_50_ values (hereafter, EC_50_ values are always determined by the Elispot assay). Testing 32 clones we found that the hillslope ranged from 0.37 to 1.86, showing that they differed up to about 5 fold (Supplementary Table 1). Furthermore, we found that the Elispot hillslopes and EC_50_ values did not correlate (Fig. 2d), supporting the notion that these two measurements represent two independent functional properties of the clones. Stability and reproducibility of the assessment of parameters of hillslope and EC_50_ using the Elispot assay are shown in Supplementary Fig. 2a and b, respectively.

### Hillslope determined with Elispot versus cytotoxicity assays

We wondered whether a similar heterogeneity assessment might be suitable by using the cytotoxicity assay instead of the Elispot assay. Therefore, we used the Hill function with variable slope to analyze the data obtained with the cytotoxicity assay. Supplementary Fig. 3a shows three representative clones with different functional avidities as assessed by the cytotoxicity assay. However, when comparing the hillslope data obtained with the two functional assays, there was only a trend but no significant correlation (Supplementary Fig. 3b). Similar to the Elispot assay, the EC_50_ and hillslope values did not correlate (Supplementary Fig. 3c). The discrepancies were unlikely caused by technical flaws because the results were reproducible (Supplementary Fig. 4a and b). Our data suggest that the hillslopes obtained with the two assays do not provide similar information. As further outlined in the discussion, an important difference between the two methods is that the cytotoxicity assay provides only indirect information about the T cells, whereas the Elispot assay delivers one value directly from each individual T cell, making it more suitable to study the T cell’s functional avidity heterogeneity.

### Hillslope: a new parameter of functional avidity heterogeneity

To verify the hypothesis that the Elispot hillslope represents the heterogeneity of functional avidity, we tested whether mixing two clones with different mean functional avidities (different EC_50_ values) would result in a curve with a lower hillslope as compared to the hillslopes of the two individual clones. As expected, the 1:1 mixture of two clones of well-defined mean functional avidities produced a titration curve with an EC_50_ value in between the high and the low avidity clone used in the mixture (Supplementary Fig. 5a). As predicted, the hillslope of the mixture was lower than the hillslopes of the two clones. To verify this observation in large numbers of clones, we asked the question whether this type of result could be obtained systematically, whenever two clones were mixed, provided that the individual clones had relatively high hillslopes and were thus presumable relatively homogenous. Therefore we grouped the clones in two categories, the homogenous clones with a hillslope above the median (0.95) and the heterogeneous clones below the median (Supplementary Table 1). We found a significant drop of the hillslopes in mixtures of clones with a high hillslope and with a relatively large EC_50_ difference, as measured and calculated previously (Supplementary Fig. 5b). In contrast, mixtures of clones with a low hillslope (Supplementary Fig. 5c) or all clones together (Supplementary Fig. 5d) did not show a significant hillslope drop. Together, these data can be taken as proof of principle that the hillslope indeed represents the functional avidity heterogeneity of the T cells.

### Biphasic titration curves with mixtures of two clones

**Figure 3:**
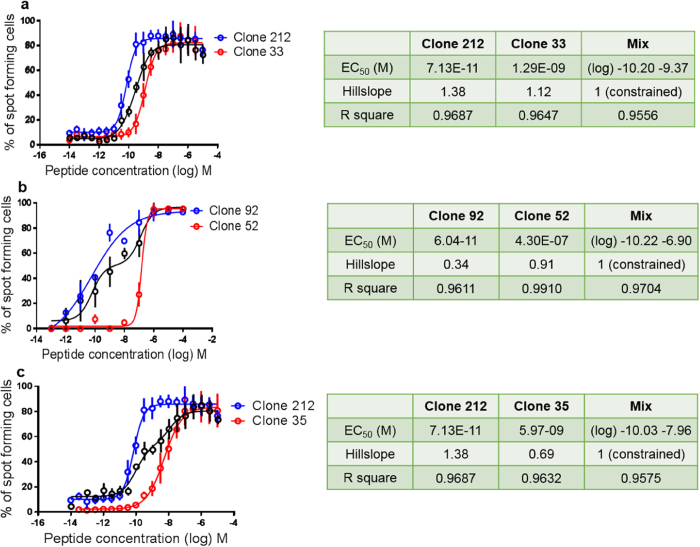
Mixtures of 2 homogenous clones with a big EC_50_ difference permits visualization and mathematical verification of a biphasic sigmoidal shape. (**a**) The postulated biphasic sigmoidal double-S curve was not apparent when we mixed clones with small EC_50_ difference, as shown with clone 212 plus clone 33. (**b**) In contrast, a double-S curve is evident in a mixture of clones with high EC_50_ difference, e.g. with clone 92 plus clone 52, and (**c**) clone 212 plus clone 35. The data from the three experiments with mixed clones were also analyzed with the classical “Hill function with variable slope” (not shown), resulting in R square values of 0.9548 (top, mix of clones 212 & 33), 0.8568 (middle, mix of clones 92 & 52) and 0.8408 (bottom, mix of clones 212 & 35).

On theoretical grounds, we hypothesized that peptide titrations with mixtures of two clones should result in biphasic sigmoid (double-S) curves, based on the rational that each clone should generate one sigmoid curve, and that this could be visualized with mixtures of two carefully selected clones. As previously, we thought that this would be best seen when one clone had a low and the other clone a high EC_50_ value, and when using homogenous clones, i.e. those with high (steep) hillslope values, as determined in single clone assays. Furthermore, using 3× versus 10× peptide titration steps and using quadruplicates resulted in better fits of the sigmoid curves (see Methods). As predicted, we found that the double-S curve was not apparent when we mixed clones with a small EC_50_ difference, as shown for the mixture of clone 212 with clone 33 (Fig. 3a). On the contrary, the expected double-S in the mixing curve was visible with mixed clones with a large EC_50_ difference, as shown for the high avidity clone 92 mixed with the low avidity clone 52 (Fig. 3b), or for the high avidity clone 212 mixed with the low avidity clone 35 (Fig. 3c). Subsequently, we aimed to determine whether a double-S curve could be modeled mathematically. We tested a simple, general, biologically reasonable modeling approach leading to a mathematical model that combines log-logistic function: one for the upslope and one for the downslope of the “biphasic” curve when combining a high with a low avidity clone (see Methods). The parameters employed are meaningfully interpretable. This model can be used to test the presence of biphasic effects, possibly also in the cases where no biphasic effect can be visually observed. We found that this model gave favorable R square values, a measure of the goodness of curve fit (Fig. 3). In contrast, the R square values obtained with a standard single-S function were less favorable (legend to Fig. 3), as predicted for the conditions in Fig. 3b and c, indicating that the data points fitted better to a double-S than to a single-S curve of the Hill function with variable slope.

### Functional avidity heterogeneity of polyclonal (total antigen specific) T cell populations

**Figure 4:**
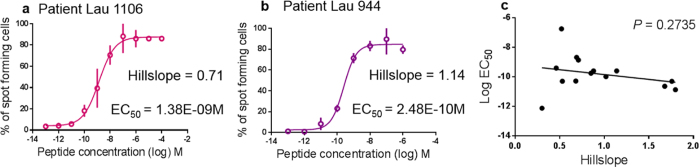
EC_50_ and hillslopes from the patient’s polyclonal Melan-A specific T cells. Two representative examples (n = 14) of assessing functional avidity of melanoma patients’ Melan-A tetramer + polyclonal T cell populations after 14 days of IVS, using the Elispot assay. (**a**) 300 Melan-A specific CD8 T cells from patient Lau 1106 displaying a shallow hillslope (0.71). (**b**) A more homogenous functional avidity was found in the Melan-A specific CD8 T cells from patient Lau 944. (C) EC_50_ and hillslope are independent values.

The T cell clones of this study were derived from melanoma patients, and from their polyclonal T cell populations specific for HLA-A2/Melan-A (hereafter called polyclonal or “total” population). Thus, the material from our patients enabled us to study both clones and total populations. As a first step “from monoclonal to polyclonal”, we had mixed two clones and showed that this may result in a significant increase of functional avidity heterogeneity. Consequently, we hypothesized that natural polyclonal populations from patients may display more heterogeneous functional avidities as compared to the clones. However, this was not always the case. The hillslopes differed considerably, with some populations displaying high heterogeneity (hillslope: 0.70, Fig. 4a), while others were remarkably homogenous (hillslope: 1.14, Fig. 4b), with hillslope values that were higher than many of the clones. As in clones, hillslope and EC_50_ did not correlate, confirming that they represent two independent parameters also for polyclonal populations (Fig. 4c). Interestingly, the range of hillslopes was from 0.30 to 1.80, comparable to the clones (Supplementary Table 2), revealing the surprising result that the functional avidity of clones is not necessarily more homogenous than the functional avidity of polyclonal populations.

### Direct comparison of individual clones with their parental populations

**Figure 5:**
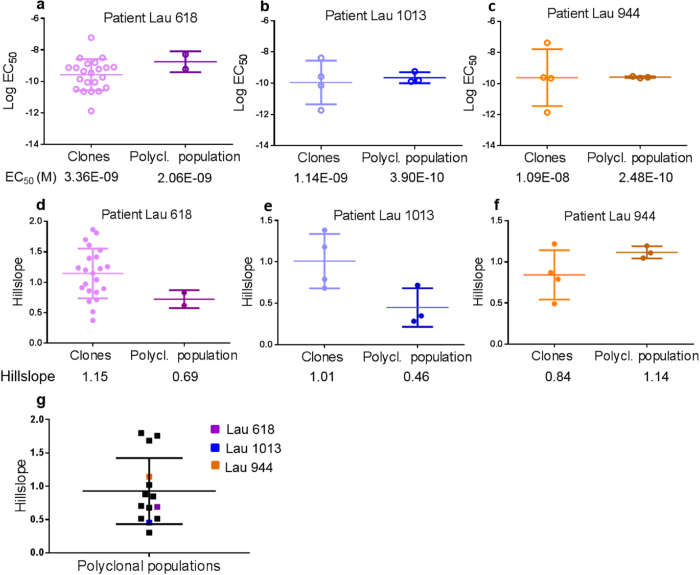
Functional avidity and its heterogeneity of clones and polyclonal populations specific for HLA-A2/Melan-A. (**a**–**c**) The average value of EC_50_ of the clones from patients Lau 618, Lau 1013 and Lau 944 was compared to the EC_50_ of the parental polyclonal populations, i.e. the populations from which the clones were derived. (**d**–**f**) The average value of the hillslopes was higher for the clones of patients Lau 618 and Lau 1013 than for the parental polyclonal populations. This was different for patient Lau 944, where the polyclonal population had a higher hillslope value than the average hillslope of its clones. The three data points for the polyclonal populations shown in (**a**–**f**) are the values from the triplicate Elispot cultures. (**g**) Compiled hillslope data of the polyclonal populations from the 14 patients. The corresponding values are shown in Supplementary Table 2.

It is well known that poly-clonal populations include several different clonotypes characterized by different TCRs, with different affinities mediating different functional avidities^22^,^36^,^37^. To directly set the results of clones in relation to their parental polyclonal populations, we analyzed the data from three different patients, namely patients Lau 618, Lau 1013 and Lau 944. First, we studied the Elispot EC_50_ values. The average functional avidities of the example clones were slightly different from the functional avidity of the parental polyclonal populations (Fig. 5a–c), as expected since we did not have all the clones needed to fully represent the polyclonal populations. In patients Lau 618 and Lau 1013 the average hillslope of the clones was higher than the hillslope of the total populations (Fig. 5d,e), compatible with the expectation that the polyclonal populations display more heterogeneous functional avidities. However, the polyclonal population of patient Lau 944 was surprisingly homogenous, particularly in view that some of its clones were heterogeneous (Fig. 5f).

### Functional avidity heterogeneity of the polyclonal T cell populations from 14 patients

Finally, we compiled the Elispot hillslope values from the 14 polyclonal T cell populations analyzed in this study (Fig. 5g). Interestingly, we discovered that 3 of the polyclonal populations had surprisingly even higher hillslope values than patient Lau 944, indicating that they were remarkably homogenous. In turn, many T cell clones were noteworthy heterogeneous. Note that that the cells used for Fig. 5a–f cannot be directly compared, because the clones do not sufficiently represent the polyclonal populations. Nevertheless, these data illustrate that the functional avidity heterogeneity in clones can be surprisingly high, and remarkably low in natural antigen specific populations. Although the majority of the polyclonal populations studied appeared heterogeneous, there are a few displaying a rather homogenous profile, possibly due to the dominance of some of their homogenous clones in their repertoires.

## Discussion

One of the main goals of cancer immunotherapy is to efficiently induce powerful tumor antigen specific CD8 T cell responses. The aim of the present study was to advance the frontiers for evaluating T cell responses, particularly with respect to T cell functional avidity. In cancer patients, functional avidity monitoring is likely useful, because the avidity of tumor antigen specific T cells may often be insufficient. Vaccination with peptide, CpG and IFA can induce high frequencies of peptide specific CD8 T cells, allowing in depth characterization of T cell responses^38^,^39^,^40^. For the present study we used previously described T cell clones generated from PBMCs withdrawn from the vaccinated patients^39^,^40^,^41^. In agreement with previous studies^42^,^43^, we confirm that the mean functional avidity, classically determined by the ^51^Chromium release cytotoxicity assay^28^,^44^, could also be efficiently measured by the IFN-γ Elispot assay.

We used the Elispot assay primarily because it provides direct information of T cells, i.e. data from the function (IFN-γ production) of each T cell. In contrast, the ^51^Cr release cytotoxicity assay, the predominantly used assay to assess functional avidity, determines the percentages of target cells killed. However, such data provide only indirect information on T cell function. There is evidence that the killing activity may largely differ between individual T cells, and that “super killers” may dominate in cytotoxicity assays^45^, meaning that many other T cells do not contribute significantly to target cell killing. For characterization of T cells and their degree of heterogeneity, it is much preferable to have direct information of T cells, with similar data weight for each individual cell. Therefore, an assay that directly analyzes T cells is likely superior.

This aspect is also important with regard to the percentages of non-functional cells. Obviously, functional avidity data can only be obtained from functional cells. In contrast to the ^51^Cr release assay, the Elispot assay delivers this information, provided that the numbers of specific cells are known. In our assays, we always used 300 specific T cells, most of which generated spots, at least at high peptide concentrations. However, some cells did not spot, i.e. they remained non-functional despite expressing a specific TCR. Besides lack of function, one may also consider the amount of cytokine produced per cell. The Elispot assay is based on counting spots that fulfill certain criteria (e.g. size, optical density), reflecting a critical threshold corresponding to a defined level of functional intensity. Therefore, this assay is well suited to interrogate the function of individual T cells. Similar goals can be achieved by other methods, e.g. by intracellular cytokine staining and flow cytometry, but the Elispot assay can be more easily extended to larger numbers of titration steps and multiple replicates.

Titrating antigenic peptide typically results in a characteristic sigmoid dose response curve, fitted by the usually applied Hill function with fixed slope. The peptide concentration that confers 50% maximal activity represents the Keff 50 or EC_50_ value, defining the mean “functional avidity”^42^,^46^. For this study we hypothesized that the titration curve would provide further information, namely that the slope of the curve would reflect the functional avidity heterogeneity. However, the usually applied Eq. (1) does not discriminate different slopes, as the applied function always constrains the slopes to equal 1.0. Therefore, we used a Hill function with variable slope which determines the “hillslope” reflecting the steepness of the curve. This allowed us to determine the slopes obtained from different T cells, revealing a large functional avidity heterogeneity spectrum with hillslopes ranging from about 0.3 to 1.8. As expected, there was no correlation of hillslopes and EC_50_ values, confirming that the hillslope represents an independent value of T cell functionality.

For the peptide titration assay, we used a minimum of nine steps of 10-fold peptide dilutions (in quadruplicates). However, using 3-fold instead of 10-fold titration steps and including higher and lower peptide concentrations to cover a broad concentration range improved the quality of the curve, with better description of the bottom and top plateaus, and a more precise S curve. Consequently, the curve fitting was improved (see Methods). As reported by others, titration curves can provide information of cellular functions that may be complex, varying in efficacy of response. Parameters within the titration curve, such as the hillslope can describe additional biological properties^47^. Here we report for the first time that the hillslope of the peptide titration curve describes the degree of functional avidity heterogeneity.

Our experiments with mixtures of two clones were designed to obtain proofs of principle. Even though these results have no practical use, they confirm the theoretical predictions. We found that the curves from experiments with mixing of two clones may reveal lower hillslopes, provided that (i) each of the two clones show high hillslopes when tested separately, and (ii) the difference of the mean functional avidities of the two clones is large. We reasoned that in this situation, the mixtures likely result in low hillslopes, because mixing two clones with very different avidity should result in high degree of functional avidity heterogeneity. Indeed, we confirmed this hypothesis. As expected and trivial, the EC_50_ from the mixing experiments showed values that were intermediate to the ones of the two clones.

On similar grounds, we postulated that the curve of a mixed population of a high plus a low functional avidity clone should have a double-S shape. Our results reveal the postulated biphasic curve, and demonstrate a good fit to the mathematical modeling of such a curve. However, this can only be shown in selected experimental conditions (i.e. when using mixtures of two clones with large EC_50_ difference, and with relatively steep hillslopes), which we used for the proof of principle. It is likely that this approach has no immediate practical use. Theoretically, one may argue that mixtures of multiple clones or even natural polyclonal T cell populations should give shapes of curves with multiple S, in a stairway like fashion, and that this can be mathematically modeled and matched with real results. However, for practical application of such an approach one needs high precision data that may often not be in reach.

Our strategy to first analyze monoclonal cells, followed by the investigation of bi-clonal populations, was pursued in view of our aim to characterize the functional avidity heterogeneity of the patients’ natural polyclonal T cell populations. Therefore, the logical next step was to perform Elispot titration experiments with the latter and compare the results with those obtained from the clones that had been generated from some of those polyclonal populations. The average Elispot EC_50_ of the clones per patient was relatively close to the Elispot EC_50_ of the parental total population, suggesting that the latter was relatively well represented by the clones. However, this was incomplete - a full representation would have required extended cloning, identification of all involved TCRs, and the full characterization of clonotypes and their degree of dominance. This was beyond the scope of this study.

In 2 of the 3 patients analyzed, the hillslope values of the total polyclonal populations were lower than the mean hillslope of the clones, corresponding to the expectation of higher functional avidity heterogeneity in the total cells as opposed to the clones^33^,^48^. However, the contrary was observed for patient Lau 944 who displayed a surprisingly high homogeneity of the total population despite some more heterogeneous clones. Further studies are necessary to elucidate the underlying mechanisms; in this patient one could for example postulate dominant homogenous clonotypes that explain the result of the total population despite presence of some minor (i.e. non-dominant) clonotypes with heterogeneous functional avidity. Our analysis revealed that the total populations of 3 of 14 patients were remarkably homogenous, whereas the remaining ones were more or clearly heterogeneous. Thus, polyclonality does not always mean high functional avidity heterogeneity. In turn, mono-clonal T cell populations often display remarkably high functional avidity heterogeneity, demonstrating that TCR-independent mechanisms may substantially impact on functional avidity.

Modulations of signaling molecules such as lck or ZAP70 have been shown to impact on functional avidity^49^,^50^,^51^. In addition, surface co-receptors may play significant roles, as discovered for PD-1 shown to modulate T cell avidity within an antigen specific repertoire^52^. Also, TCR multimerization and membrane lipids are functionally relevant. Increased sizes of oligomeric TCR complexes lead to increased antigen sensitivity of memory T cells^53^. Interestingly, TCR dimerization occurs independently of the antigen and appears to be mediated by cholesterol and sphingomyelin binding to the TCRβ chain^54^. An important role of cholesterol metabolism was recently demonstrated by pharmacological inhibition of ACAT1, a key cholesterol esterification enzyme, leading to enhanced effector functions and proliferation of CD8 T cells^55^. Finally, functional avidity is strongly dependent on the T cell’s cycle and activation states. Indeed, many of the above mentioned parameters vary depending on the degree of T cell activation. Despite all these parameters, TCR affinity remains a major determinant, and may also impact on heterogeneity, as increased affinity will directly result in reduced heterogeneity^56^.

T cell responses may be associated with maturation of functional avidity^57^. Changes can either occur via clonal remodeling, i.e. changes in clonotypic distribution involving different TCRs^58^,^59^,^60^, or TCR-independently due to mechanisms mentioned above^49^,^50^,^51^. Despite that our study does not represent a progress for the identification of mechanisms regulating functional avidity, we think that assessing heterogeneity is worthwhile. For example, it will be useful to determine whether maturation of functional avidity involves reduction of its heterogeneity. It might also be interesting to know whether the spectrum and heterogeneity of functional avidity correlates with protection from infectious diseases or cancer, or induction of autoimmune diseases or allograft rejection. Finally, avidity modulation may have implications for immunotherapy, as different methods to promote the patient’s T cell responses could result in changed functional avidity due to clonal selection and/or modification of TCR-independent functional avidity mechanisms^61^.

Many fundamental questions remain open. Further research is required to determine whether the IFN-γ response is digital or analog. Several studies have shown that T cells respond by IFN-γ production in a digital manner, i.e. that individual T cells respond or not upon antigen encounter^62^,^63^. However, other studies provided evidence that the response is analog, i.e. that individual T cells produce IFN-γ depending on antigen concentration and off rates of TCR-pMHC binding^56^,^64^. It might be possible that both apply, in the sense that the response is basically digital, but with an additional analog component which is proportional to the rate of signaling despite that intracellular signaling is based on digital events^56^.

In summary, we found that the IFN-γ Elispot assay allows to determine the functional avidity heterogeneity, represented by the steepness/hillslope of the peptide titration curve. T cell clones can show low but also surprisingly high functional avidity heterogeneity. Future studies will advance the characterization of T cell functional avidity in immune responses, progressively promoting the understanding of the importance and the regulation of T cell avidity.

## Additional Information

**How to cite this article:** Ioannidou, K. *et al*. Heterogeneity assessment of functional T cell avidity. *Sci. Rep.* **7**, 44320; doi: 10.1038/srep44320 (2017).

**Publisher's note:** Springer Nature remains neutral with regard to jurisdictional claims in published maps and institutional affiliations.

## Supplementary Material

Supplementary Information
