# Supplementary material for: Heterogeneity assessment of functional T cell avidity
Source: Sci Rep. 2017 Mar 13;7:44320. doi: 10.1038/srep44320 (PMC5347081; doi:10.1038/srep44320)
Supplement: Supplementary Information [file srep44320-s1.pdf]

SUPPLEMENTARY INFORMATION

Heterogeneity assessment of functional T cell avidity

Kalliopi Ioannidou, Petra Baumgaertner, Philippe O. Gannon, Michel F. Speiser, Mathilde Allard, Michael Hebeisen, Nathalie Rufer and Daniel E. Speiser

Supplementary Figures page 1
Supplementary Tables page 5
Supplementary Methods page 7
Supplementary References page 9

Supplementary Figures

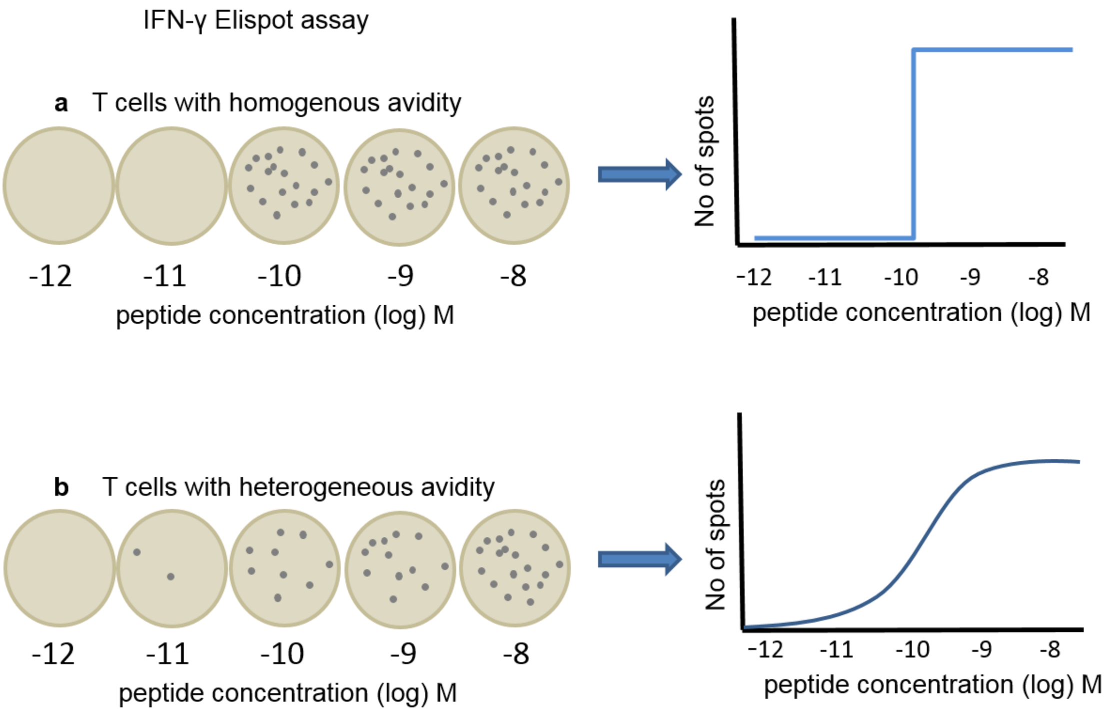

Supplementary Figure 1|Theoretical representation of low vs. high functional avidity heterogeneity and their hypothetical titration curves. (**a**) A theoretical completely homogenous clone consisting of cells that all spot at exactly the same peptide concentration, resulting in a vertical curve. (**b**) The slope becomes shallower, when the cells respond with different peptide sensitivity, revealing heterogeneity.

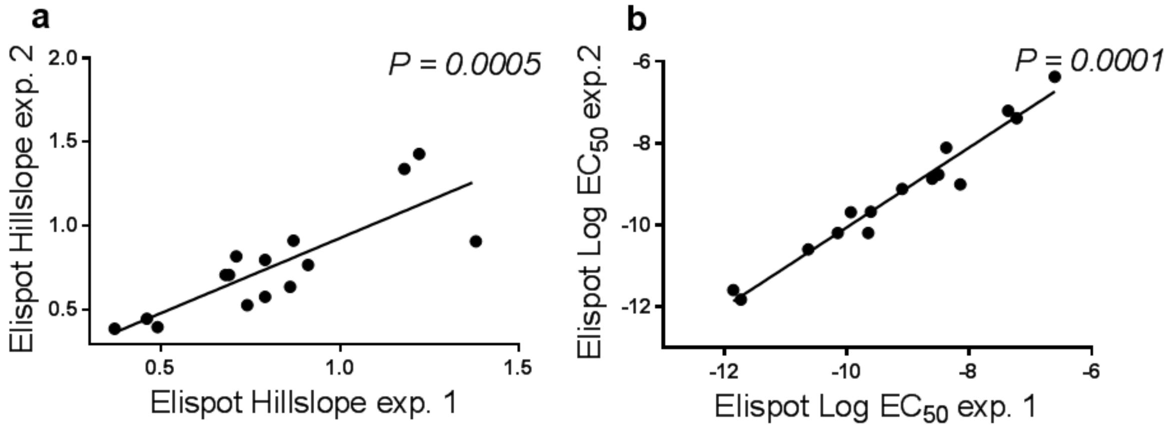

Supplementary Figure 2|Reproducibility of hillslope (**a**) and EC_{50} (**b**) values obtained from 15 T cell clones, assessed by the IFN-γ Elispot assay.

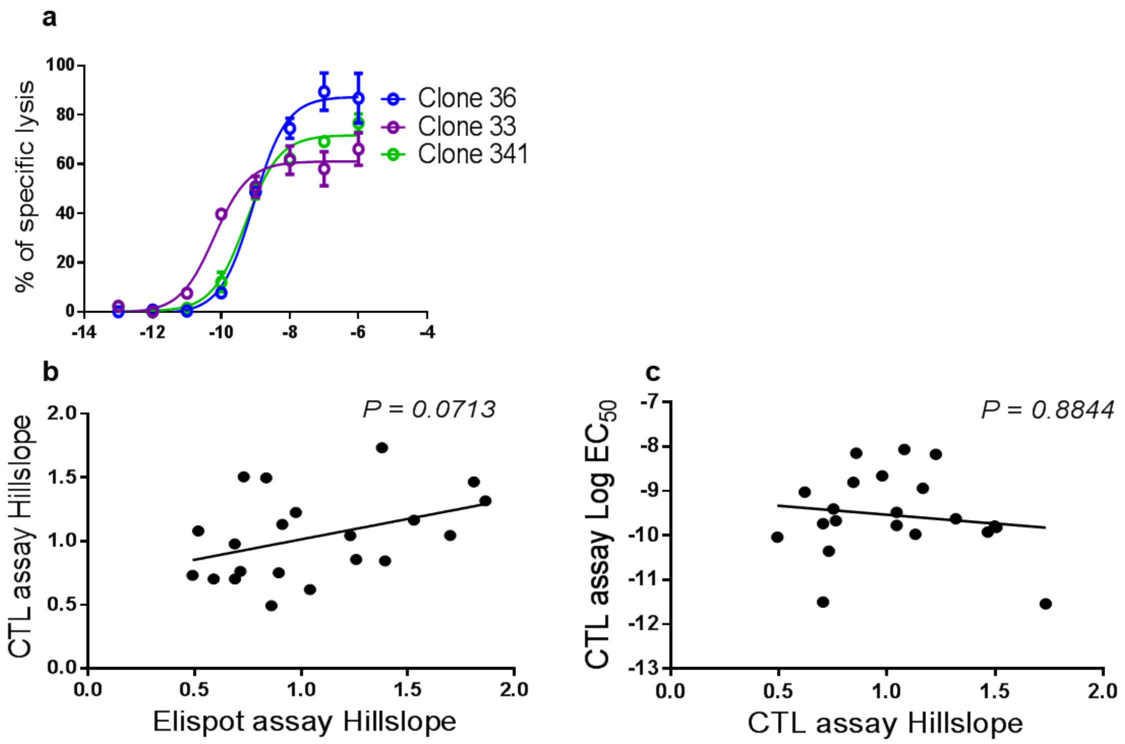

Supplementary Figure 3| (**a**) Cytotoxicity assay data from 3 different clones using the Hill function with variable slope. (**b**) Comparison of hillslopes assessed by the Hill function with variable slope, using the Elispot and the cytotoxicity assay. (**c**) EC_{50} and hillslope using the cytotoxicity assay do not correlate.

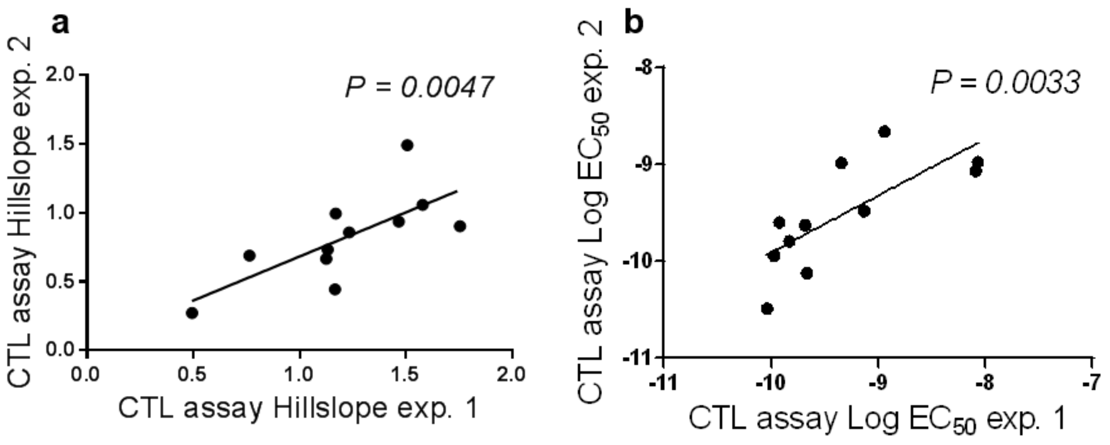

Supplementary Figure 4|Reproducibility of hillslope (**a**) and EC_{50} (**b**) values obtained from 11 T cell clones, assessed by the cytotoxicity assay.

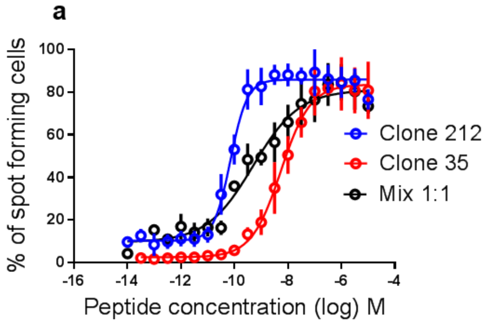

Clones	Clone 212	Clone 35	Mix
EC_{50} (M)	7.13E-11	5.97E-09	4.96E-10
Hillslope	1.38	0.69	0.50
R square	0.9687	0.9632	0.9320

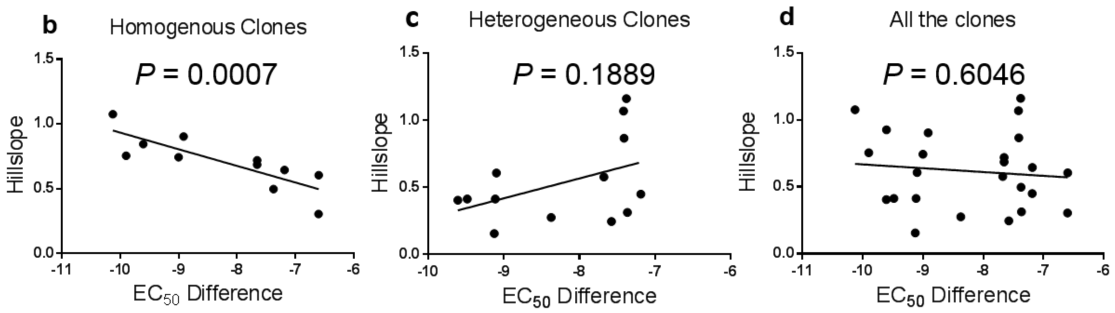

Supplementary Figure 5|**Elispot peptide titration curves of a clone with high and a clone with low mean functional avidity, respectively, and the mixture thereof.** (**a**) 300 cells were plated for each of the two clones (blue and red curves, respectively), and a mixture of 150 cells of the high functional avidity clone with 150 cells of the low functional avidity clone (black curve). As predicted, the black curve shows an intermediate EC_{50} value, and a lower hillslope than the ones of the two individual clones. Data are representative for 3 independent experiments. (**b-d**) Elispot peptide titration experiments with mixed populations of 2 homogenous or 2 heterogeneous clones, in relation to the EC_{50} differences of the two clones used for the experiment**.** (**b**) Significant correlation of the hillslope with the EC_{50} difference in experiments with 2 homogenous clones. Lack of significance when using (**c**) 2 heterogeneous clones, or (**d**) any clones (compilation of all data). All values were determined with the Elispot assay.

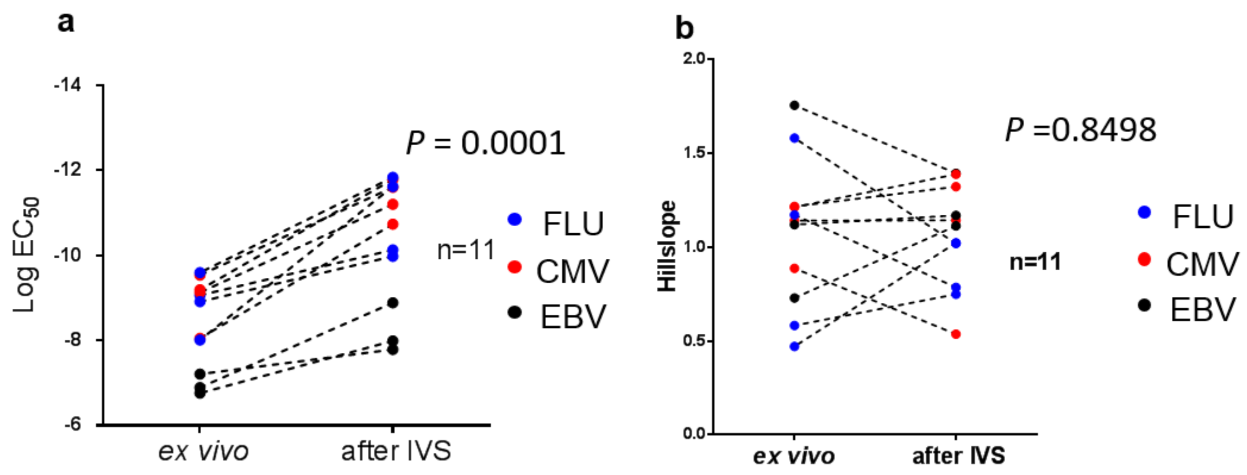

Supplementary Figure 6|Comparison of IFN-γ Elispot data obtained from T cells directly assessed directly *ex vivo* versus after in vitro stimulation (IVS) for expansion during 12 days. (**a**) EC_{50} values. (**b**) Hillslope values. Polyclonal CD8 T cell populations specific for CMV, EBV or FLU viral antigens were obtained from 11 healthy donors.

Supplementary Tables

Patient (Lau)	Patient (short nr)	Clone	EC_{50} (M)	Hillslope	R Square	Max no of spots
618	3	36	8.06E-10	0.37	0.9390	148
944	9	92	2.25E-10	0.49	0.9071	209
618	3	312	7.58E-10	0.52	0.9918	179
975	20	201	1.80E-12	0.59	0.9533	163
1013	21	214	2.71E-10	0.68	0.9708	155
972	11	111	2.49E-09	0.69	0.9184	158
618	3	35	5.97E-08	0.69	0.9455	66
618	3	32	3.15E-09	0.71	0.9697	160
371	5	51	1.17E-10	0.73	0.9315	110
1013	21	211	1.86E-12	0.79	0.9428	238
944	9	91	1.40E-12	0.79	0.9093	197
618	3	34	2.35E-11	0.86	0.9006	196
944	9	94	4.32E-08	0.87	0.9768	127
618	3	319	3.94E-11	0.89	0.8397	176
618	3	317	2.24E-11	0.91	0.7184	198
371	5	52	2.51E-07	0.91	0.9828	139
618	3	316	6.02E-10	0.97	0.9780	144
618	3	314	1.33E-12	1.04	0.9123	154
618	3	340	4.43E-10	1.15	0.9838	173
1013	21	213	4.23E-09	1.18	0.9651	233
618	3	343	7.35E-10	1.20	0.9545	196
944	9	93	2.48E-10	1.22	0.9840	185
618	3	33	1.33E-09	1.23	0.9643	297
618	3	37	8.78E-11	1.24	0.9851	341
618	3	347	7.42E-10	1.26	0.9702	154
1013	21	212	7.13E-11	1.38	0.9687	289
618	3	311	3.06E-09	1.39	0.8974	176
618	3	344	1.54E-09	1.42	0.9881	175
618	3	346	3.49E-10	1.53	0.9713	206
618	3	310	2.28E-11	1.70	0.9805	167
618	3	342	1.84E-10	1.81	0.9672	221
618	3	341	1.39E-10	1.86	0.9747	223

Supplementary Table 1 | Overview of all T cell clones tested by the IFN-γ Elispot assay. EC_{50}, Hillslope, R square (as a measure of the goodness of curve fit) and maximal numbers of spots were determined by Elispot assays as described in Methods.

Patient (Lau)	No. of vaccinations	% t ELA+ *ex vivo*	% t ELA+ *in vitro*	EC_{50} (M)	Hillslope	R square	Days after IVS	Max no of spots
444	20	0.68	17.7	7.37E-13	0.30	0.7430	12	186
1013	8	0.81	N/A	3.90E-10	0.46	0.9145	15	222
706	4	0.13	4.45	1.77E-07	0.52	0.6262	15	120
1264	22	0.68	12.3	4.97E-11	0.53	0.9072	12	102
1090	16	0.06	36.8	5.16E-11	0.68	0.7153	11	54
618	20	0.9	13.6	2.06E-09	0.69	0.9335	13	206
1106	25	0.75	42.5	1.38E-09	0.71	0.9591	14	121
392	7	0.36	22.6	1.75E-10	0.85	0.9422	12	342
1022	8	0.32	9.37	2.49E-10	0.88	0.9334	12	330
1164	10	3.48	55.4	1.03E-10	1.02	0.9222	11	328
944	16	1.43	58	2.48E-10	1.14	0.9802	14	120
972	20	3.48	79.7	2.29E-11	1.68	0.9667	12	140
672	4	0.12	19.3	4.87E-11	1.76	0.9869	12	375
648	12	0.17	17.8	1.33E-11	1.80	0.8202	11	100

Supplementary Table 2|Analysis of total polyclonal HLA-A2/Melan-A specific T cell populations. No of vaccinations, number of monthly vaccinations with Melan-A peptide, CpG 7909 and IFA that the patients had received before taking blood for T cell analysis. Frequencies of A2/Melan-A tetramer+ (t ELA+) cells before (*in vivo*) and after *in vitro* stimulation (IVS) for 12 days, determined by flow cytometry with fluorescent tetramers. EC_{50}, Hillslope, R square and maximal numbers of spots were determined by Elispot assays as described in Methods.

Supplementary Methods

Blood withdrawal. Blood samples were taken one week after vaccination. Lymphoprep™ (Fresenius Kabi Norge AS, 12HGS05) centrifuged PBMCs were cryopreserved in RPMI 1640, 40% FCS, and 10% DMSO at a concentration of $10x10^6$ PBMCs per ml for 72 h at -80°C and then transferred into the vapor phase of liquid nitrogen until further use.

Purification of CD8 T cells. $10x10^6$ PBMCs were thawed, washed, and resuspended with 80 µl cell sorting buffer (PBS, 0.2 % BSA, 5 µM EDTA), kept on ice. 20 µl of CD8 microbeads (Miltenyi, 130-045-201) were added, mixed and incubated for 30 min (mixing every 10 min) in the fridge. Cells were washed by adding 2 ml of cell sorting buffer, centrifuged at 1500 rpm for 5 minutes and resuspended in 500 µl of cell sorting buffer. MS columns (Miltenyi) were loaded with a maximal number of 10^7 labeled cells or $2x10^8$ total cells. 15 ml Falcon tubes were labeled as CD8- and placed below the columns to collect the flow-through of unlabeled CD8- cells. The columns were rinsed once with 500 µl of cell sorting buffer, before the addition of the cell suspension. The volume passed through entirely and the column was washed three times by adding 500 µl of cell sorting buffer. The columns were removed from the separators and placed onto Falcon tubes. 2 ml of cell sorting buffer was added to the columns and the plunger was pushed firmly into the columns to flush the positive-labeled cells. The CD8- and the CD8+ cells fractions were centrifuged and washed after with 2 ml RPMI medium supplemented with 8% Human Serum to determine the cell number.

Expansion of CD8 T cell clones. Upon expansion, the specificity of each generated T cell clone was verified by multimer staining. T cell clones were expanded by periodic (every 14–21 d) restimulation with PHA, human IL-2, and irradiated feeder cells. Monoclonality was confirmed for the majority of the clones used in this study, by sequencing of TCR-α and -β chains, and by FACS staining with Vβ specific antibodies (data not shown).

***In vitro* stimulation (IVS) of purified CD8 T cells with peptide for expansion of the antigen specific T cells**. Because the available blood volume and cell numbers from melanoma patients were low, we first expanded them by *in vitro* stimulation (IVS) for 12-14 days. The CD8- fraction was irradiated with a dose of 30 Grays and resuspended with fresh RPMI + 8% Human Serum. Melan-A analog peptide (ELAGIGILTV, stock 1mg/ml), Cytomegalovirus peptide (CMV; NLVPMVATV, stock 1mg/ml), Epstein–Barr virus peptide (EBV; GLCTLVAML, stock 1mg/ml), and Influenza virus peptide (FLU; GILGFVFTL, stock 1mg/ml), were added respectively, at the final concentration of 1 µM. CD8 T cells were added and incubated at 37°C. After 24 hours, IL-2 was added, starting with 50 U/ml, and increased to 100 U/ml on the second day, and from the next day on, with 150 U/ml. The culture medium was changed twice per week. After 12-14 days, the antigen specificity was determined by tetramer FACS staining, and cultures were used for Elispot assays. We studied the impact of *in vitro* stimulation (IVS) on the functional avidity and heterogeneity of the expanded cells, in comparison to direct *ex vivo* analysis. Analyzing 11 healthy donors with different specificities for CMV, EBV and FLU virus we found that the EC_{50} values in all cases were significantly increased (**Supplementary Fig. 6a**). Therefore, IVS increased the functional avidity of the expanded cells, likely through enhanced activation and co-receptor activity [1-3]. In contrast, the hillslope values stayed similar or changed to variable degrees, such that the difference between direct *ex vivo* analysis and after IVS was not statistically significant (**Supplementary Fig. 6b**), with some populations showing increased and others

decreased hillslopes after IVS. Overall, IVS clearly impacted on the T cell's avidity, suggesting that future studies should preferably be done directly *ex vivo*, albeit this requires large blood volumes.

Chromium release cytotoxicity assay. Chromium release assays were performed using radioactive ^{51}Chromium. For peptide titration experiments, ^{51}Cr-labeled TAP2/2-deficient T2 cells (HLA-A*0201+) were pulsed with serial dilutions from 10^{-6} M to 10^{-13} M of Melan-A EAAGIGILTV peptide. The effector cell to target cell (E:T) ratio was 10:1. After 4 hours incubation, supernatants were analyzed in a TopCount NXT benchtop microplate scintillation and luminescence counter. The percentage of specific lysis was calculated as 100 x (experimental - spontaneous release) / (total - spontaneous release). The cytotoxicity assays were performed three times.

IFN-γ Elispot assay. 25 µl of 35% ethanol/well was put in 96 well PVDF plates (MSIPS4510, Millipore) and incubated at room temperature (RT) for 30 seconds. The wells were emptied by flicking the plate over a sink and gently tapping on absorbent paper. The plates were thoroughly washed 3x with 100 µl 1X PBS per well. 100 µl of diluted capture antibody (100 µl into 10 ml 1X PBS) was added to every well and the plates were covered and incubated at 4°C overnight. The wells were washed as previously, once with 100 µl 1X PBS. 100 µl of culture medium with 10% serum was added to every well and the plates were incubated at RT for 2 hours. The wells were washed with 100 µl 1X PBS, and 300 antigen specific CD8 T cells in 50 µl of RPMI with 8% Human serum were plated with 50 µl of 20'000 T2 cells per well and the addition of 1 µM of the native Melan A peptide (EAA, stock 1mg/ml), in a total volume of 200 µl per well. The plates were incubated at 37°C in a CO_2 incubator for 20 hours. With the aim to always plate 300 antigen specific T cells per well, the polyclonal T cell populations were first characterized with fluorescent tetramers by flow cytometry, and the numbers of cells were adjusted according to the percent of tetramer-positive T cells. The next day the plates were incubated with 100 µl of 0.05% PBS-Tween solution (wash buffer) per well at 4°C for 10 min and subsequently 3x with wash buffer. 100 µl of diluted detection antibody (100 µl into 10 ml Dilution buffer) was added to every well and incubated at RT for 1 hour 30 min. The plates were washed 3x with 100 µl of wash buffer and 100 µl of diluted Streptavidin –AP conjugate was added to every well. After an incubation of 1 hour at RT, the plates were washed 3x with wash buffer and 3x with distilled water (both sides of the membrane, peeling off the plate bottom). 100 µl of BCIP/NBT buffer was added to every well and the plates were incubated for 5-15 min, monitoring spot formation visually throughout the incubation to assess sufficient color development. The wells were emptied and both sides of the membrane were rinsed 3x with distilled water. The frequency of the resulting colored spots corresponding to the cytokine producing cells, was determined using the Elispot Bioreader 5000. All Elispot experiments were performed in triplicates. For clones, the experiments were performed 2-3 times. The total polyclonal populations were only tested once because of the limited cell availability. The standard deviations from the triplicate Elispot cultures (**Figure 5a-f**) were relatively small. Furthermore, we always tested a clone of a well-known functional avidity as a control in parallel to the patients' polyclonal populations, and only used the results from those experiments in which the controls showed proper reproducibility.

Supplementary References

1 Goff, S. L. *et al.* Enhanced receptor expression and in vitro effector function of a murine-human hybrid MART-1-reactive T cell receptor following a rapid expansion. *Cancer Immunol. Immunother.* **59**, 1551-1560 (2010).

2 Lee, K. H. *et al.* Increased vaccine-specific T cell frequency after peptide-based vaccination correlates with increased susceptibility to in vitro stimulation but does not lead to tumor regression. *J. Immunol.* **163**, 6292-6300 (1999).

3 Montes, M. *et al.* Optimum in vitro expansion of human antigen-specific CD8 T cells for adoptive transfer therapy. *Clin. Exp. Immunol.* **142**, 292-302 (2005).
